# Supplementary material for: Characterizing cell-type spatial relationships across length scales in spatially resolved omics data
Source: Nat Commun. 2025 Jan 3;16:350. doi: 10.1038/s41467-024-55700-1 (PMC11699133; doi:10.1038/s41467-024-55700-1)
Supplement: Supplementary file 3 — Supplementary Software 1 [file 41467_2024_55700_MOESM3_ESM.zip › CRAWDAD/plots/slideseq/S3_slideseq_bergmann_trends_all.pdf]

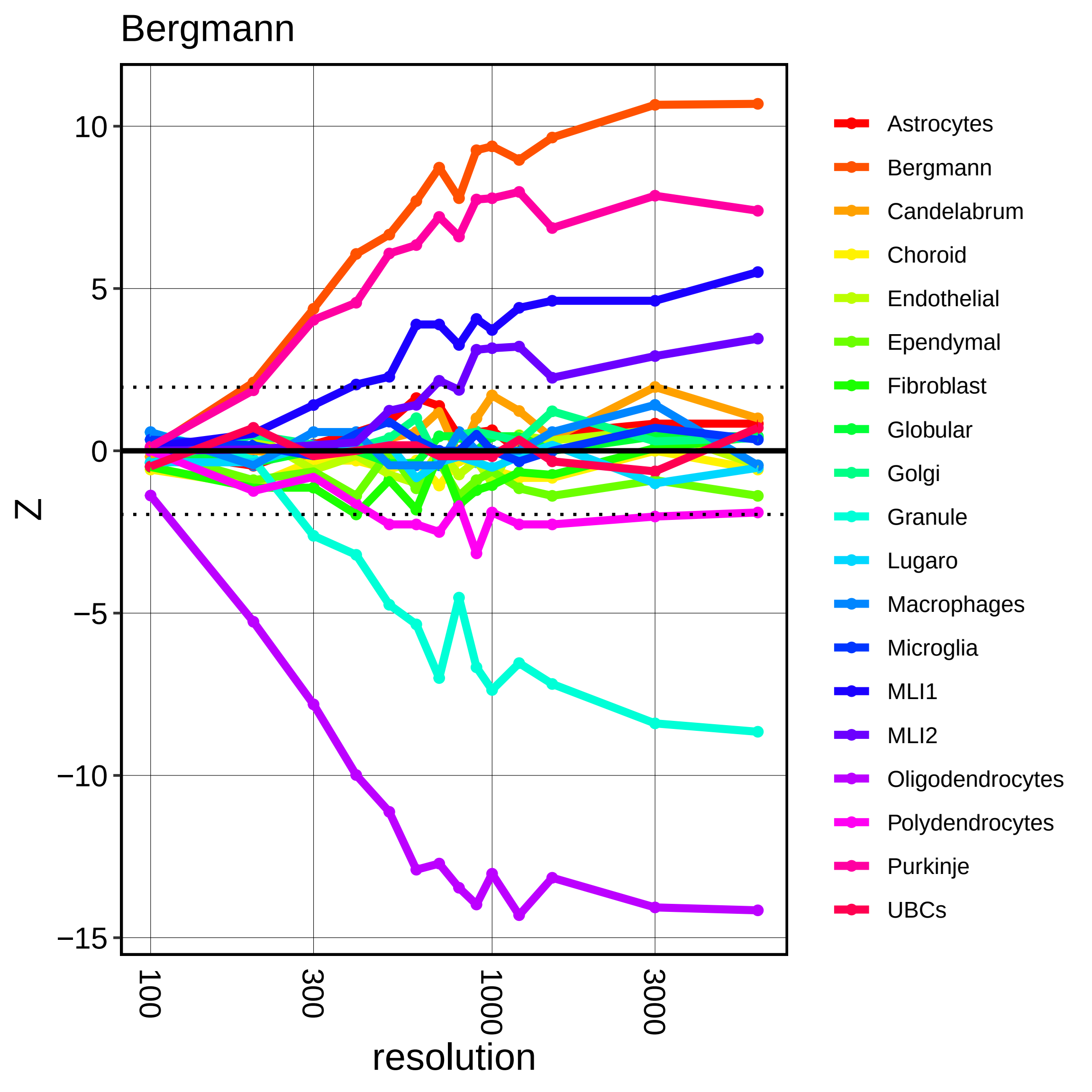
Bergmann
10
5
0
−5
−10
−15
Z
100
300
1000
3000
resolution
Astrocytes
Bergmann
Candelabrum
Choroid
Endothelial
Ependymal
Fibroblast
Globular
Golgi
Granule
Lugaro
Macrophages
Microglia
MLI1
MLI2
Oligodendrocytes
Polydendrocytes
Purkinje
UBCs
